# Monetarism and the General Practitioner

**Published:** 1990-03

**Authors:** M. J. Whitfield


### From our correspondents

**MONETARISM AND THE GENERAL PRACTITIONER**

The government has decided that its monetarist policy should extend into the management of the NHS. In 'Working for Patients' the government outlines its policy which, in essence, is a blueprint for controlling the ever-escalating costs of health care. It seeks to exert this control by a two pronged budgetary approach, one at regional level and one at the basic provider level.

The regional budgetary control is very similar to that in existence at present, except that it encompasses the whole of national health care, not simply that provided by the district health authorities. The Family Practitioner Committee services are being put under the authority of the region and budgetary control will extend into prescribing, staff and rent reimbursements and, ultimately, into the cost of referral to specialist services.

The other budgetary control that is proposed is new and introduces a potentially damaging principle concerning our current health service. General practices of over 9,000 patients are being encouraged to accept budgets of their own. When a region accepts a practice as a budget holding practice, the practice will be allocated a sum of money out of which it must provide all NHS prescriptions for their patients, all NHS investigations such as pathological and radiological investigations and the costs of the NHS referrals made to specialists. The only exclusions to this are those referrals made 'as emergencies'. These budgets will require constant monitoring and computer software is being manufactured which will enable each practice to determine how its expenses are going. This second system of budgetary control can be seen to have a number of advantages as far as cost control is concerned. The provider (general practitioner) will be intimately involved in the costs of health care and will be forced to make judgements about the value of certain procedures. The GP will be encouraged to consider whether a prescription or an investigation or referral is crucial for good patient care. Is it essential for a patient to have an investigation to check that all is now well, or should that investigation not be performed, thus saving some of the practice budget? The evidence that those rural doctors who dispense their own prescriptions have considerably lower prescribing costs than their colleagues who simply write prescriptions that are dispensed by a pharmacist, supports those who claim that having a financial interest in providing care will force the provider to control costs.

In an attempt to ensure that standards of care don't fall the government is making great play of medical audit, suggesting that this will ensure that standards of care will be maintained by this mechanism, together with encouraging patients to change their doctor if they perceive their care is less than optimal.

What are my concerns about these proposals? They are based on my experience of medical practice in the USA and Australia, which have very different systems of health care, but which have lessons for us to learn. Both these countries commit a much greater proportion of their wealth to health care than we do. Firstly, most of the general practitioners in the NHS are proud of this present system of care, although most regret it is under-resourced compared to most other developed countries. If the proposals for change are instituted, the financial implications of care and **the opportunities to manipulate general practice budgets for personal gain**, will inevitably result in some entrepreneurial GPs putting their personal profit before good patient care.

Let me give an example of how this can happen. Un-used profits from practice budgets may be used to 'improve practices', not for personal gain, say the regulations. If these profits are put into surgery premises or pay for extra members of staff, this will enable the GPs to retain more of their practice profit for their own use. I see no way that this abuse of budget funds can be effectively policed and see this as the major draw back to these proposals. Naturally, GPs who become budget-holders will see the advantages and will be tempted to use these as a way of increasing their personal income in the same way as do dispensing doctors at present.

My second concern about the new proposals is the **administrative cost** that is involved. This can be divided into two parts, central cost and practice administration cost.

Central administrative costs are rising astronomically. General managers are replacing administrators, financial directors are replacing finance officers and the salaries for each are increasing by at least 50%. The numbers of staff employed by FPCs will need to increase in order to manage the budgets of GPs, service audit committees, liaise with region and so on. The information technology required is probably unattainable in the time available. Certianly, accurate costing of referrals and investigations is unlikely to be available by April 1991.

Practice administrative costs too are inevitably rising. Our own FPC has a 50% increase this year in its ancillary staff budget. Most of this will go towards paying the new practice managers (many of whom are commanding salaries of between £15,000–20,000 per annum). Other new staff are being appointed to run practice computers which will be needed to manage the practice budgets. If only a proportion of these costs had been made available for patient care . . .

My third area of concern is **the introduction of a new system of care without a pilot study**. The government claims that no general practitioner is being forced to volunteer to become a budget holder, and that those that do volunteer will effectively be trying out the system. The bribes being offered to those practices that become budget holders involve large sums of money as administrative costs, so many practices will feel they would be foolish to refuse the opportunity. At present we have no effective ways of measuring quality of care. Changing a system of medical care to such a significant degree without having a method of measuring whether care is improved or deteriorates seems very foolish. Finally I am concerned about the proposals for those **practices that decide not to become budget holders**. The purchaser units of health authorities are to negotiate contracts with hospitals to provide services such as specific operations at specified cost, and outpatient services at specified cost, I fear that this will result in considerably reduced choice for those practices for these type of patient service.

The new proposals for change in general practice are scheduled to begin in about a year's time. If the goverment retains it's present timetable, this country's general practitioner services are about to take a great stride into the unknown. It will certainly be in the direction of a heavily cost-conscious service and well away from that service that most of the current generation of general practitioners have been proud to be associated with. Many of us who have experienced medical care systems in other countries are very concerned that many of the advantages patients have in this country will be lost following the introduction of this new system. Linking the cost of medical care to the potential income of the provider, although theoretically attractive to a monetarist, may well reduce the standards of primary care of NHS patients.

M. J. WHITFIELD

